# Kimura's Disease

**DOI:** 10.1155/2009/424053

**Published:** 2009-06-11

**Authors:** Cláudia Savassi Guimaraes, Natalie Moulton-Levy, Allen Sapadin, Claudia Vidal

**Affiliations:** Department of Dermatology, Mount Sinai School of Medicine, New York, NY 10029, USA

## Abstract

Kimuras disease is a chronic inflammatory disorder of unknown etiology. It is rare in the West, but endemic in Asia. It typically presents as solitary or multiple subcutaneous nodules, that slowly increase in size. The lesions are variably painful and pruritic. It often accompanied by regional lymphadenopathy, raised serum eosinophil counts, and markedly elevated serum immunoglobulin E levels. Histologically, the lesions are characterized by reactive lymphoid follicles with eosinophilic infiltration and an increased amount of postcapillary venules. The optimal treatment for KD remains controversial. Although the condition seldom resolves spontaneously, malignant transformation has not been reported to date, and the prognosis is good. We describe a male patient with a 4-year pruritic progressive “bump” in front of his left ear. On physical examination, the patient had 2 discrete lesions on the left side of his face near his ear. Postauricularly, there was a 3 × 5 cm erythematous to violaceous, indurated nodule. Preauricularly, there was a similar, but smaller cyst-like nodule. Punch biopsy showed a superficial and deep nodular and interstitial infiltrate, reactive lymphoid follicles with a dense infiltration of eosinophils and areas of eosinophilic follicle lysis. The patient received intralesional triamcinolone acetonide injections 10 mg/cc behind left ear with a good improvement.

## 1. Introduction

Kimura's disease (KD) is a chronic inflammatory disorder of unknown etiology. It is a rare entity in the West, but endemic in Asia. The typical clinical presentation is characterized by painless, sometimes disfiguring, subcutaneous nodules, predominantly in the head and neck. It often accompanied by regional lymphadenopathy, raised serum eosinophil counts, and markedly elevated serum immunoglobulin E (IgE) levels [1, 2]. Histologically, the lesions are characterized by reactive lymphoid follicles with eosinophilic infiltration, sometimes forming eosinophilic abscesses [2–4]. The optimal treatment for KD remains controversial. Although the condition seldom resolves spontaneously, malignant transformation has not been reported to date, and the prognosis is good. Still, early diagnosis of KD could spare the patient unnecessary and potential harmful diagnostic procedures.

## 2. Report of a Case

A 62-year-old, male presents with an extremely pruritic “bump” in front of his left ear. He reported it was initially flat and had progressively increased in size over 4 years. He denied pain and other associated symptoms. His medical, surgical, and family histories were noncontributory. He was not taking medications, and he had no drug allergies. He reported trying multiple topical therapies for his condition without improvement. Interestingly, he reported some improvement with “allergy pills”. The patient was born in the Dominican Republic and immigrated to the United States several years prior to presentation. He frequently travels back to his native country and last traveled there approximately 1 year prior. The patient is employed as a janitor in an apartment building in the Bronx. He denied tobacco, alcohol, or drug use.

**Figure 1:**
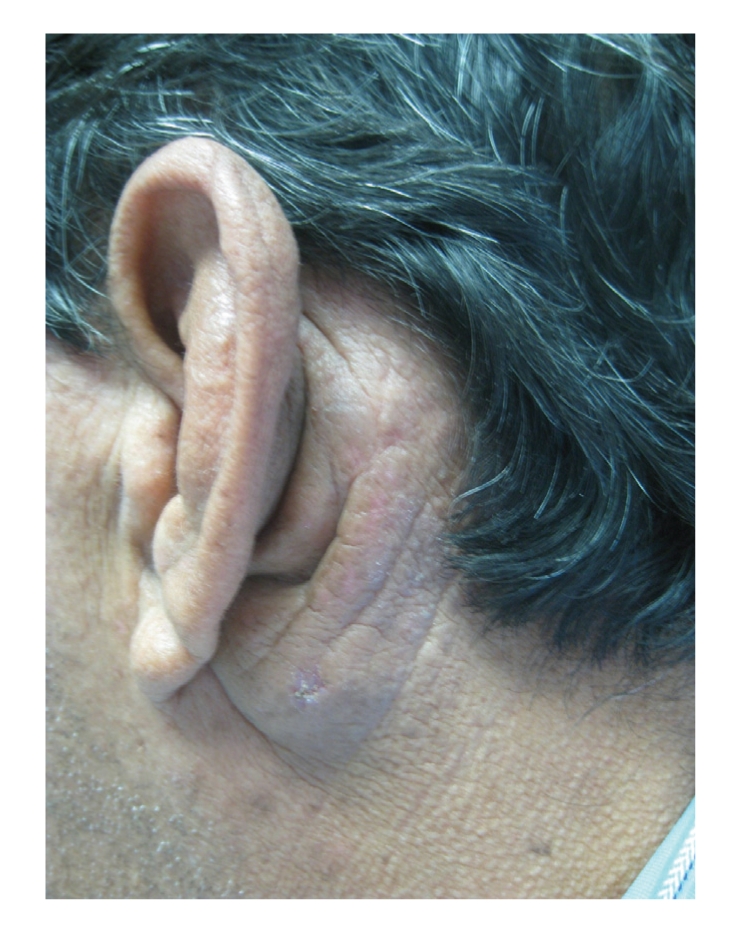
Clinical appearence.

**Figure 2:**
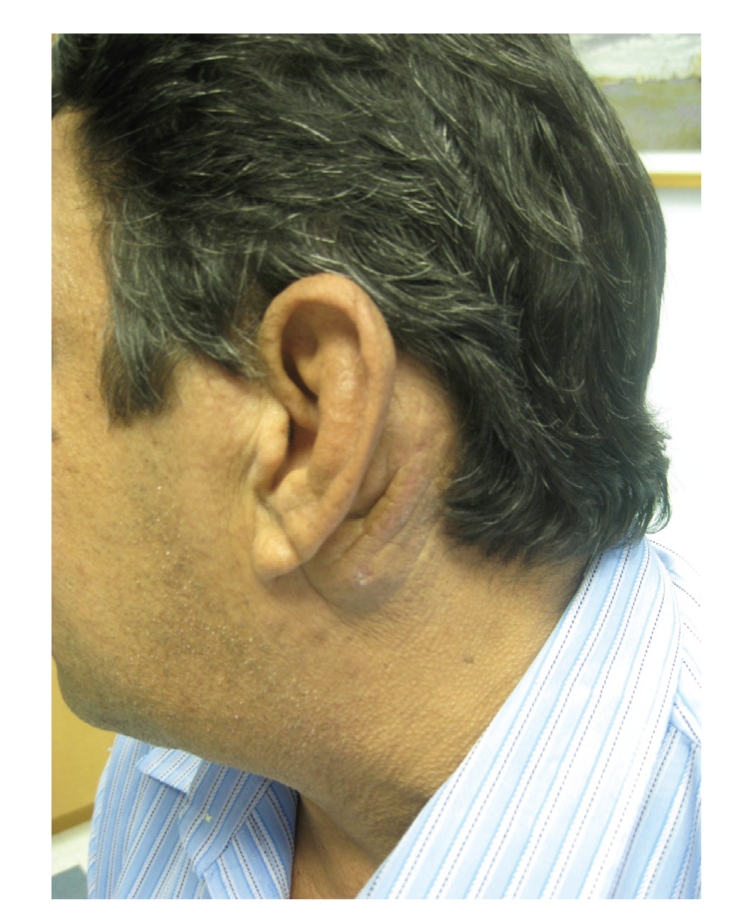
Clinical appearance.

On physical examination, the patient had 2 discrete lesions on the left side of his face near his ear. Postauricularly, there was a 5 × 3 cm erythematous to violaceous, indurated nodule. Preauricularly, there was a similar, but smaller cyst-like nodule. (Figures 1  and 2). There were no lesions on the external ear, and no pharyngeal lesions were appreciated. Laboratory values were significant for a white blood cell (WBC) count of 9.6/10^3^ *μ*L (reference range: 4.5–10.5/10^3^ *μ*L), with an eosinophil count of 902 cells/mcL (reference range: 15–550 cells/mcL). His hemoglobin level was 16.4 g/dL (reference range: 13.8–17.2 g/dL) with a hematocrit of 49.3% (reference range: 41–50%). Serum IgE level was 1647 KU/L (reference range: ≤114 KU/L). Glucose, sodium, potassium, chloride, carbon dioxide, urea nitrogen, creatinine, calcium, protein, and liver enzyme levels were all within normal limits.

**Figure 3:**
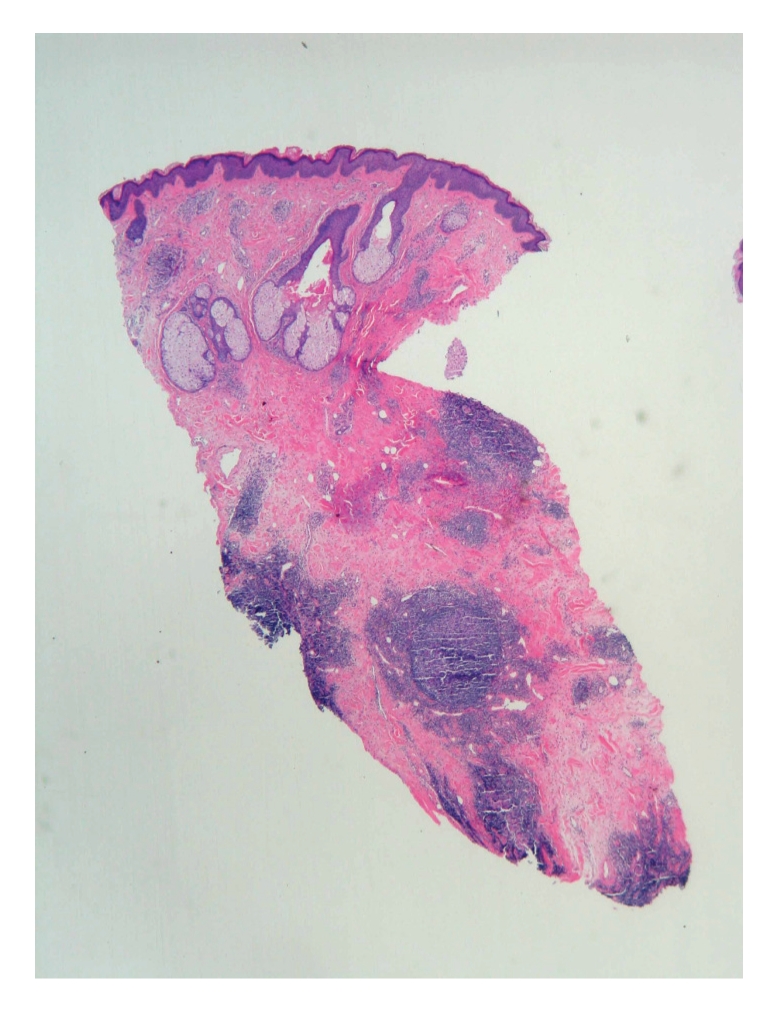
Histology.

**Figure 4:**
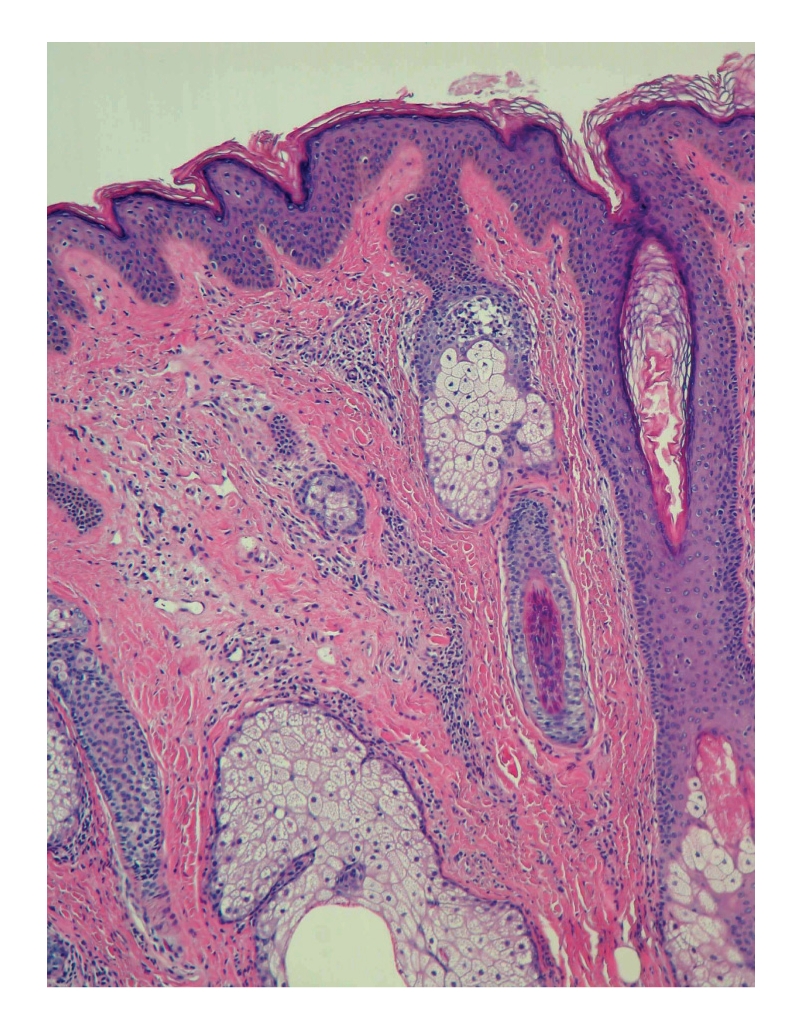
Histology.

**Figure 5:**
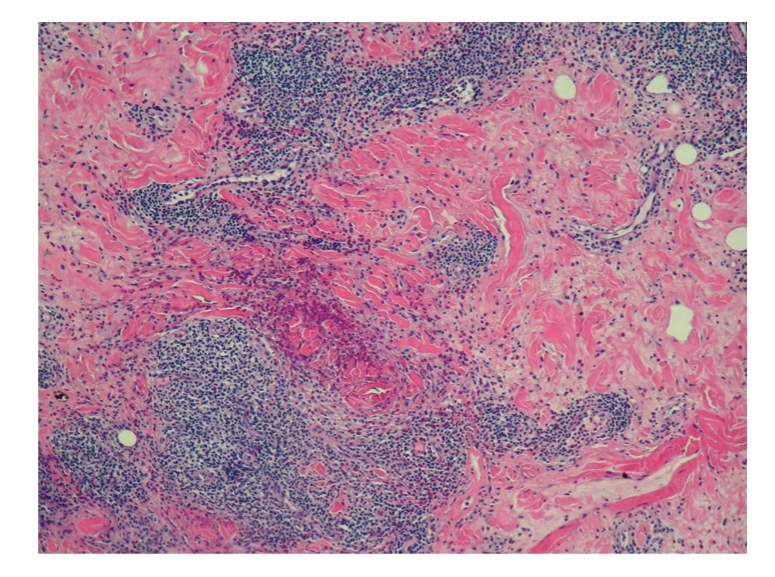
Histology.

**Figure 6:**
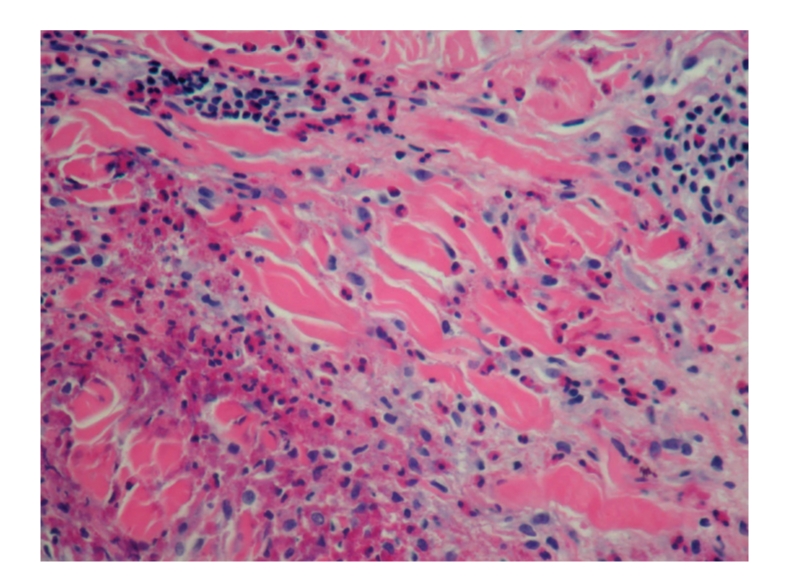
Histology.

**Figure 7:**
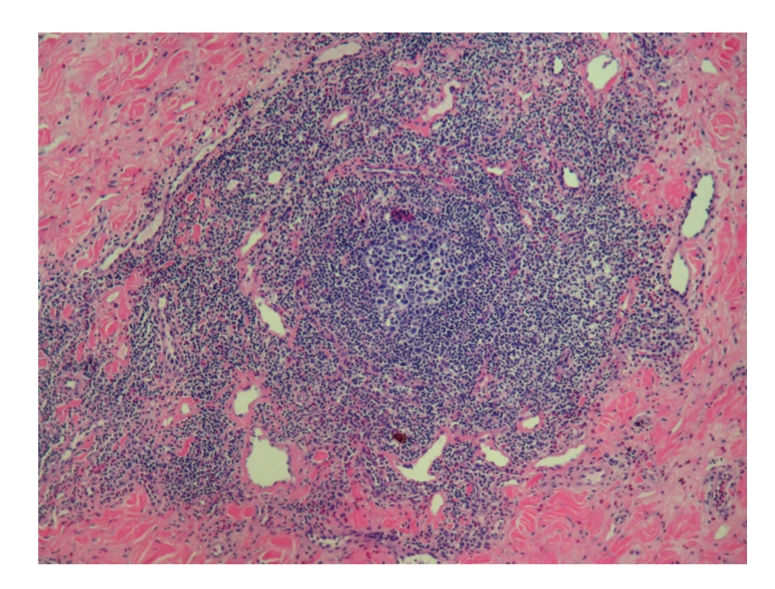
Histology.

**Figure 8:**
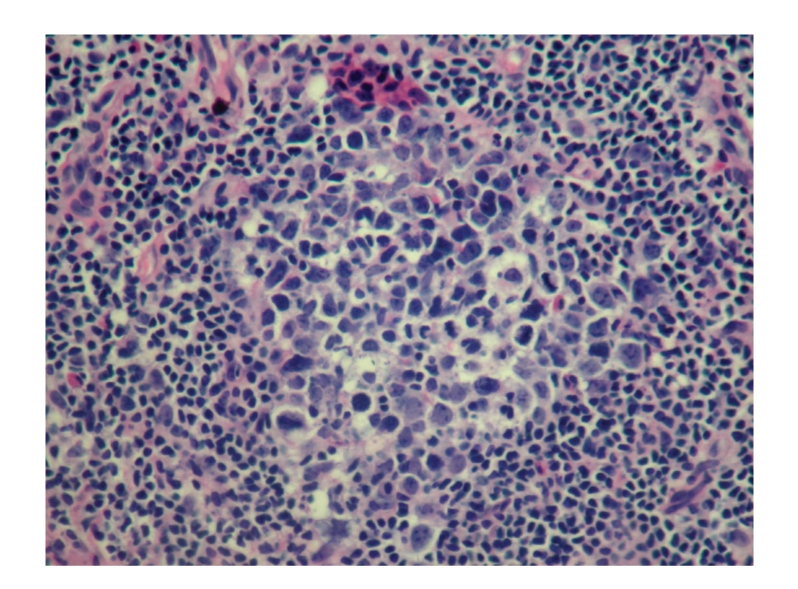
Histology.

**Figure 9:**
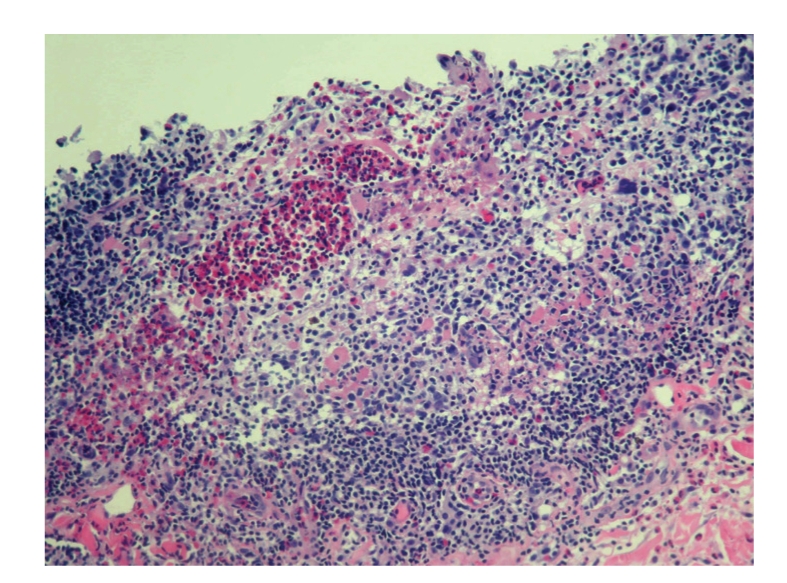
Histology.

**Figure 10:**
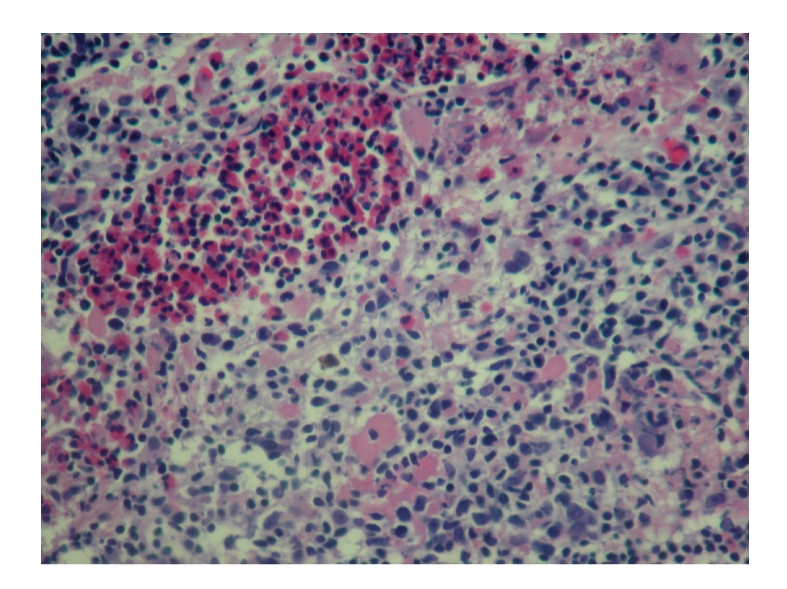
Histology.

Punch biopsy of the postauricular lesion showed a superficial and deep nodular and interstitial infiltrate. There were numerous reactive lymphoid follicles with a dense infiltration of eosinophils and areas of eosinophilic follicle lysis. Additionally, eosinophilic granules encrusted on collagen (flame figures) were found secondary to the interstitial eosinophils. The vessels were increased in number with notable flat endothelial cells (Figures 3, 4, 5, 6, 7, 8, 9, 10).

All figures are for a patient that received intralesional triamcinolone acetonide injections 10 mg/cc behind left ear with a good improvement.

## 3. Discussion

Kimura's disease (KD) is a rare entity in the west, but endemic in Asia. It is a benign chronic inflammatory disease with unknown etiology although a possible relation to Epstein Bar virus (EBV) infection has been suggested [5]. This entity was first described in China by Kim and Szeto [6] in 1937. However, it did not become widely recognized until 1948 when Kimura and colleagues described cases in Japan by their histologic findings of “unusual granulation combined with hyperplastic changes in lymphoid tissue”. Subsequently, this disorder came to be known as Kimura's disease. KD typically affects patients in their second to third decades of life, with the median age of affected individuals being 28 years old in one series. There is a male predominance, with six times as many males being affected than females. The prevalence is unknown, but the majority of reports describe cases from Asian countries, such as China, Japan, Taiwan, and Hong Kong. Nonetheless, sporadic cases do occur in other ethnic groups. A case series of patients from the US demonstrated that persons of any racial group may be affected and have clinical and histological features indistinguishable from those patients from Asia.

The clinical presentation is variable. It typically presents as solitary or multiple subcutaneous nodules, that slowly increase in size. The lesions are variably painful and pruritic; however, the overlying skin is normal. The most common site of involvement is the cervical region [7]. However, it may occur in many other sites including the limbs, groin, trunk, and scalp. Regional lymphadenopathy is often present [1, 2, 8]. Associations with asthma, Loeffler's syndrome, and connective tissue disease have rarely been reported. The frequency of nephrotic syndrome in these patients may be up to 60%, a frequency well exceeding that of the general population [9–11]. An examination of the urine should be performed, as proteinuria suggests an associated nephritic. Laboratory findings include peripheral eosinophilia and elevated serum IgE levels. 

Definitive diagnosis of KD is based on histologic findings. In 1989, the various histologic features of Kimura's disease were classified as being constant, frequent and rare. The constant features are preserved lymph node architecture, florid germinal centers, eosinophilic infiltration, and an increased amount of postcapillary venules. Cutaneous eosinophilic vasculitis has been described [7]. The frequent features include sclerosis, karyocytosis in both the germinal centers and the paracortex, vascularization of the germinal centers, proteinaceous deposits in germinal centers, eosinophilic abscesses, and atrophic venules in sclerotic areas. The single rare feature is the progressive transformation of germinal centers [12]. Immunochemical findings are IgE reticular network in germinal centers and IgE-coated nondegranulated mast cells [4]. Histologic findings differentiate Kimura's disease from more common causes of head and neck tumors.

The clinical differential diagnosis includes both benign and malignant processes, including salivary gland tumor, nodal metastasis, lymphoma, reactive lymphadenopathy, and Mikulicz's disease [1, 2]. The absence of Reed-Sternberg cells helps exclude Hodgkin's disease. Differentiating between KD and T-cell lymphomas can be difficult because they can both present with polymorphonuclear lymphocytes and eosinophilia. 

The main differential diagnosis is angiolymphoid hyperplasia with eosinophilia (ALHE). The diseases are clinically similar in that they are both soft tissue swellings in the head and neck area. ALHE is more typically found as dermal nodules in middle-aged women as opposed to the deeper subcutaneous nodules of KD, which predominantly affect young men. In addition, lymphadenopathy is rare in AHLE. Histologically, both disorders are characterized by vascular proliferation and lymphoid infiltration with eosinophils. In ALHE, there are vascular endothelial cells prominent thick walled-vessels with vascular endothelial cells of exhibiting nuclei of varied size and shape and hemosiderin deposits. These changes are not seen in KD. These histologic changes suggest that ALHE is neoplastic in origin, whereas KD is an immune-mediated disorder [4, 13].

Kimura's disease is a medically benign disorder, which is typically chronic, although spontaneous resolution has been reported. There is no consensus about the optimal treatment for KD. Therapy should aim to preserve cosmesis and function while preventing recurrences and long term sequelae. Some authors consider surgical excision to be first-line therapy; although relapses are common [7]. Topical and systemic corticosteroids are frequently used, but tumors may become refractory to treatment. In refractory lesions, or in patients who are not candidates for more conventional methods of treatment, local radiation therapy (25–30 Gy) has been used. Sequelae from irradiation, like xerostomia, must be considered. Other treatment options include systemic or intralesional cytotoxic agents and intralesional steroids. The combination of all-trans-Retinoic acid with low dose of prednisone successfully treated one patient with KD. In two cases, pranlukast, a leukotriene receptor antagonist, (450 mg/day) was reported to be effective treatment for KD, without any adverse side effects. In a corticosteroid-dependent patient, treatment with etirizine induced a complete remission within 2 months. Despite steroid discontinuation, this patient remained disease-free at follow-up 6 months later [7]. 

Although Kimura's disease has no known malignant potential, this disorder may clinically mimic a number of malignant tumors of the head and neck. It is important that clinicians recognize this entity, so that prompt and accurate diagnosis can be made, sparing the patient any unnecessary and potentially harmful diagnostic procedures.
